# An AUC-based permutation variable importance measure for random forests

**DOI:** 10.1186/1471-2105-14-119

**Published:** 2013-04-05

**Authors:** Silke Janitza, Carolin Strobl, Anne-Laure Boulesteix

**Affiliations:** 1 Department of Medical Informatics, Biometry and Epidemiology, University of Munich, Marchioninistr. 15, D-81377, Munich, Germany; 2 Department of Psychology, University of Zurich, Binzmühlestr. 14, Zurich, CH-8050, Switzerland

**Keywords:** Random forest, Conditional inference trees, Variable importance measure, Feature selection, Unbalanced data, Class imbalance, Area under the curve.

## Abstract

**Background:**

The random forest (RF) method is a commonly used tool for classification with high dimensional data as well as for ranking candidate predictors based on the so-called random forest variable importance measures (VIMs). However the classification performance of RF is known to be suboptimal in case of strongly unbalanced data, i.e. data where response class sizes differ considerably. Suggestions were made to obtain better classification performance based either on sampling procedures or on cost sensitivity analyses. However to our knowledge the performance of the VIMs has not yet been examined in the case of unbalanced response classes. In this paper we explore the performance of the permutation VIM for unbalanced data settings and introduce an alternative permutation VIM based on the area under the curve (AUC) that is expected to be more robust towards class imbalance.

**Results:**

We investigated the performance of the standard permutation VIM and of our novel AUC-based permutation VIM for different class imbalance levels using simulated data and real data. The results suggest that the new AUC-based permutation VIM outperforms the standard permutation VIM for unbalanced data settings while both permutation VIMs have equal performance for balanced data settings.

**Conclusions:**

The standard permutation VIM loses its ability to discriminate between associated predictors and predictors not associated with the response for increasing class imbalance. It is outperformed by our new AUC-based permutation VIM for unbalanced data settings, while the performance of both VIMs is very similar in the case of balanced classes. The new AUC-based VIM is implemented in the R package party for the unbiased RF variant based on conditional inference trees. The codes implementing our study are available from the companion website: http://www.ibe.med.uni-muenchen.de/organisation/mitarbeiter/070_drittmittel/janitza/index.html.

## Background

In bioinformatics and related fields, such as statistical genomics and genetic epidemiology, data are often highly correlated, heterogeneous and high-dimensional, with the number of predictors, also known as features or descriptors, exceeding the number of observations. The random forest (RF) approach developed by Leo Breiman in 2001 [1] is particularly appropriate to handle such complex data [2]. In bioinformatics, RF is a commonly used tool for classification or regression purposes as well as for ranking candidate predictors through its inbuilt variable importance measures (VIMs). It has been used in many applications involving high-dimensional data. As a nonparametric method RF can deal with nonlinearity, interactions, correlated predictors and heterogeneity, which makes it attractive in genetic epidemiology [3-7]. However in the context of classification, i.e. when the response to be predicted is a class membership, classification performance of RF has been shown to be suboptimal in case of strongly unbalanced data [8-10], i. e. when class sizes differ considerably.

In epidemiology, unbalanced data are observed, e.g., in population-based studies where only a small number of subjects develop a certain disease over time, while most subjects remain healthy. Unbalanced data are also common in screening studies, where most of the screened persons are negative, as well as in subclass analyses, e.g., if one wants to differentiate between different subtypes of cancer. Usually some subclasses are more common than other subclasses leading to an imbalance in class sizes. Studies on rare diseases are a further example of unbalanced data settings in medicine. Data can be obtained only from few persons having the specific rare disease, while samples from healthy control persons are much easier to obtain. Of course unbalanced data are also relevant in various other areas of application beyond the biomedical field, e.g., the prediction of creditworthiness of a bank’s costumers [11], the detection of fraudulent telephone calls [12] or the detection of oil spills in satellite radar images [13], just to name a few examples. Unbalanced data may arise whenever the class memberships are observed after data collection.

Like many other classification methods RF produces classification rules that do not accurately predict the minority class if data are unbalanced. The RF classifier allocates new observations more often to the majority class unless the difference between the classes is large and classes are well separable. For extreme class imbalances, e.g. if the minority class includes only 5% of the observations, it might happen that the RF classifier allocates every observation to the majority class independently of the predictors, yielding a minimal error rate of 5%. Although this error rate of 5% is very small, such a trivial classification is of no practical use.

Some suggestions have been made to yield a useful classification based either on sampling procedures [14-17] or on cost sensitivity analyses [14]. Sampling procedures create an artificial balance between two or more classes by oversampling the minority class and/or downsampling the majority class. Cost sensitivity analyses attribute a higher cost to the misclassification of an observation from the minority class to impede the trivial systematic classification to the larger class. Both aspects have been widely discussed in the literature with respect to RF’s classification performance [14,15,18-21]. Recent simulation studies [9] have shown that the performance of RF classification for unbalanced data depends on (i) the imbalance ratio, (ii) the class overlap and (iii) the sample size.

The impact of class imbalance on the RF VIM, however, has to our knowledge not yet been examined in the literature. In this article we focus on the permutation VIM which is known to be almost unbiased and more reliable than the Gini VIM. The latter has been shown to have a preference for certain types of predictors [22-25] and therefore its rankings have to be treated with caution. We concentrate on the class imbalance problem for two response classes with respect to the permutation VIM. We investigate the mechanisms of changes in performance for unbalanced data settings and motivate the use of a new permutation VIM which is not based on the error rate but on the area under the curve (AUC). The AUC can be seen as an accuracy measure putting the same weight on both classes – in contrast to the error rate which essentially gives more weight to the majority class. As such, the AUC is a particularly appropriate prediction accuracy measure in unbalanced data settings [26]. A permutation VIM in which the error rate is replaced by the AUC is therefore a promising alternative to the standard permutation VIM. We performed extensive simulation studies to explore and compare the behaviour of both permutation VIMs for different class imbalance levels, effect sizes and sample sizes.

## Methods

The RF algorithm is a classification and regression method often used for high-dimensional data settings where the number of predictors exceeds the number of observations. Note that throughout this article we use the term predictors which is equivalent to features or descriptors denoting variables that are used to discriminate the response classes. In the RF algorithm several individual decision trees are combined to make a final prediction. The final prediction is then the average (for regression) or the majority vote (for classification) of the predictions of all trees in the forest. Each tree is fitted to a random sample of observations (with or without replacement) from the original sample. Observations not used to construct a tree are termed out-of-bag (OOB) observations for that tree. For each split in each tree a randomly drawn subset of predictors is assessed as candidates for splitting and the predictor yielding the best split is finally chosen for the split. In the original version of RF developed by Leo Breiman [1], the selected split is the split with the largest decrease in Gini impurity. In a later version of RF, conditional inference tests are used for selecting the best split in an unbiased way [27]. For each split in a tree, each candidate predictor from the randomly drawn subset is globally tested for its association with the response, yielding a global p-value. The predictor with the smallest p-value is selected, and within this globally selected predictor the best split is finally chosen for the split.

Both forest versions implement so called variable importance measures which can be used to get a ranking of the predictors according to their association with the response. In the following, we briefly introduce the standard permutation VIM as well as our novel permutation VIM, which is based on the area under the curve.

### Random forest variable importance measures

RF’s variable importance measures are often used for feature selection for high-dimensional data settings which makes it especially attractive for bioinformatics and related fields, where identifying a subset of relevant predictors from a large set of candidate predictors is a major challenge (known as the “small n large p” problem). The two standard VIMs for feature selection with RF are the Gini VIM and the permutation VIM. Roughly speaking the Gini VIM of a predictor of interest is the sum over the forest of the decreases of Gini impurity generated by this predictor whenever it was selected for splitting, scaled by the number of trees. This measure has been shown to prefer certain types of predictors [22-25]. The resulting predictor ranking should therefore be treated with caution. That is why in this paper we focus on the permutation VIM that gives essentially unbiased error rate rankings of the predictors.

### Error-rate-based permutation VIM

From now on, we denote the standard permutation VIM as “error-rate-based permutation VIM”, since it is based on the OOB error rate, as outlined below. More precisely, it measures the difference between the OOB error rate after and before permuting the values of the predictor of interest. The error-rate-based permutation variable importance (VI) for predictor j is defined by:

(1) $${\mathrm{VI}}_{j}^{\left(\mathrm{ER}\right)}=\frac{1}{\mathrm{ntree}}\sum_{t=1}^{\mathrm{ntree}}\left(ER_{t\mathrm{j˜}}-ER_{\mathrm{tj}}\right)$$

Where

•ntree denotes the number of trees in the forest,

•ER_tj_ denotes the mean error rate over all OOB observations in tree t before permuting predictor j,

•ER_tj_ denotes the mean error rate over all OOB observations in tree t after randomly permuting predictor j.

The idea underlying this VIM is the following: If the predictor is not associated with the response, the permutation of its values has no influence on the classification, and thus also no influence on the error rate. The error rate of the forest is not substantially affected by the permutation and the VI of the predictor takes a value close to zero, indicating no association between the predictor and the response. In contrast, if response and predictor are associated, the permutation of the predictor values destroys this association. “Knocking out” this predictor by permuting its values results in a worse classification leading to an increased error rate. The difference in error rates before and after randomly permuting the predictor thus takes a positive value reflecting the high importance of this predictor.

### A novel AUC-based permutation VIM

Our new AUC-based permutation VIM is closely related to the error-rate-based permutation VIM. They only differ with respect to the prediction accuracy measure: In a nutshell, the error rate of a tree involved in (1) is replaced by the area under the curve (AUC) [28]. We define the AUC-based permutation VI for predictor j as:

(2) $${\mathrm{VI}}_{j}^{\left(\mathrm{AUC}\right)}=\frac{1}{{\mathrm{ntree}}^{*}}\sum_{t=1}^{{\mathrm{ntree}}^{*}}\left(\mathrm{AU}\;C_{\mathrm{tj}}-\mathrm{AU}\;C_{\mathrm{tj˜}}\right)$$

•ntree^∗^ denotes the number of trees in the forest whose OOB observations include observations from both classes,

•AUC_tj_ denotes the area under the curve computed from the OOB observations in tree t before permuting predictor j,

•AUC_tj_ denotes the area under the curve computed from the OOB observations in tree t after randomly permuting predictor j.

Instead of computing the error rate for each tree after and before permuting a predictor, the AUC is computed. The AUC for a tree is based on the so-called class probabilities, i.e. the estimated probability of each observation to belong to the class Y = 0 or Y = 1, respectively. The class probabilities of an observation are determined by the relative amount of training observations belonging to the corresponding class in the terminal node in which an observation falls into. If one considers an OOB observation with Y = 0 and an OOB observation with Y = 1, a “good tree” is expected to assign a larger class probability for class Y = 1 to the observation truly belonging to class Y = 1 than to the observation belonging to class Y = 0. The AUC for a tree corresponds to the proportion of pairs for which this is the case. It can be seen as an estimator of the probability that a randomly chosen observation from class Y = 1 receives a higher class probability for class Y = 1 than a randomly chosen observation from class Y = 0. Note that with the use of the AUC, the information contained in the class probabilities returned by a tree are adequately exploited. This is not the case for the error rate, that requires a dichotomization of class probabilities. From a practical point of view, the AUC is computed by making use of its equivalence with the Mann–Whitney-U statistic. The Mann–Whitney-U statistic is solely based on the rankings of two independent samples. AUC values of 1 correspond to a perfect tree classifier, since a perfect classifier would attribute each observation from one class a higher probability to belong to this class than any observation from the other class. AUC values of 0.5 correspond to a useless tree classifier that randomly allocates class probabilities to the observations. In this case in about half the cases a randomly drawn observation from one class receives a higher probability of belonging to that class than a randomly drawn observation from the other class.

The novel AUC-based permutation VIM is implemented in the package party for the unbiased RF variant based on conditional inference trees. Note that the discrepancy in performance between the standard permutation VIM and the AUC-based permutation VIM is transferable to the original version of RF since the VI ranking mechanism is completely independent from the construction of the trees.

### Comparison studies

The behavior of the two introduced permutation VIMs is expected to be different in the presence of unbalanced data. The AUC is a prediction accuracy measure which puts the same weight on both classes independently of their sizes [26]. The error rate, in contrast, gives essentially more weight to the majority class because it does not take class affiliations into account and regards all misclassifications equally important. In the results section we try to explain the consequences for the performance of the permutation VIMs for unbalanced data settings and provide evidence for our supposition. We performed studies on simulated and on real data to explore and contrast the performance of both permutation VIMs. Using simulated data we aim to see whether total sample size and effect size play a role for the class imbalance problem. We explored this by varying the total number of observations and by simulating predictors with different effect sizes. Furthermore we conducted analyses based on real data to provide additional evidence based on realistic data structures which usually incorporate complex interdependencies. Our comparison studies on simulated and on real data were conducted using the unbiased RF variant based on conditional inference trees. The implementation of this unbiased RF variant is available in the R system for statistical computing via the package party [29].

### Simulated data

The considered simulation design represents a scenario where the predictors associated with the response variable Y (binary) are to be identified from a set of continuous predictors. We performed simulations for varying imbalance levels: 50% corresponding to a completely balanced sample, 40%, 30%, 20%, 10%, 5% and 1% corresponding to different imbalance levels from slight to very extreme class imbalances. The simulation setting comprises both predictors not associated with the response and associated predictors with three different levels of effect sizes. Table 1 presents the data setting used throughout this simulation.

**Table 1:** Distribution of predictors in class 1 and class 2

**Predictors**	**Distribution in class 1**	**Distribution in class 2**	**Effect size**
X_1, …,_ X_5_	N (1.00, 1)	N (0, 1)	strong effect
X_6, …,_ X_10_	N (0.75, 1)	N (0, 1)	moderate effect
X_11, …,_ X_15_	N (0.50, 1)	N (0, 1)	weak effect
X_16, …,_ X_65_	N (0, 1)	N (0, 1)	no effect

The first five predictors X_1_, . . ., X_5_ differ strongly between classes with mean μ_1_ = 1 in one class and mean μ_2_ = 0 in the other class. The predictors X_6_, . . ., X_10_ have a moderate mean difference between the two classes with μ_1_ = 0.75 and μ_2_ = 0. For X_11_, . . ., X_15_ there is only a small difference between the classes with μ_1_ = 0.5 and μ_2_ = 0. We simulated 50 additional predictors following a standard normal distribution with no association to the response variable (termed noise predictors).

We performed analyses with varying sample sizes and report the results for total sample sizes of n = 100, n = 500 and n = 1000. For each parameter combination, i.e. imbalance level and sample size, we simulated 100 datasets and computed AUC-based and error-rate-based permutation VIs for each dataset. Note that for a sample size of n = 100 an imbalance of 1% is not meaningful since there is only one observation in the minority class.

Forest and tree parameters were held fixed. The parameter ntree denoting the number of trees in a forest was set to 1000, the parameter for the number of candidate splits mtry was set to the default value of 5. We used subsampling instead of bootstrap sampling for constructing the trees, i.e. setting the parameter replace to FALSE [22]. Conditional inference trees were grown to maximal possible depth, i.e. setting the parameters minsplit, minbucket and mincriterion in the cforest function to zero.

### Real data

We also investigated the performance of the error-rate-based and the AUC-based permutation VIM on real data including complex dependencies (e.g. correlations) and predictors of different scales. The dataset is about RNA editing in land plants [30]. RNA editing is the modification of the RNA sequence from the corresponding DNA template. It occurs e.g. in plant mitochondria where some cytidines are converted to uridines before translation (abbreviated with C-to-U conversion in the following). The dataset comprises a total of 43 predictors: 41 categorical predictors (40 nucleotides at positions −20 to 20 relative to the edited site and one predictor describing the codon position) and two continuous predictors (one for the estimated folding energy and one predictor describing the difference in estimated folding energy between pre-edited and edited sequences). It includes 2694 observations, where exactly one half has an edited site and the other half has a non-edited site. The data are publicly available from the journal’s homepage. After excluding observations with missing values, a total of 2613 observations were left, where 1307 had a non-edited site and 1306 observations had an edited site. We used this balanced dataset to explore the performance of ER- and AUC-based permutation VIM for varying class imbalances – but now with realistic dependencies and predictors of different scales. For this purpose, we artificially created different imbalance levels by drawing random subsets from the class with edited sites.

**Figure 1:**
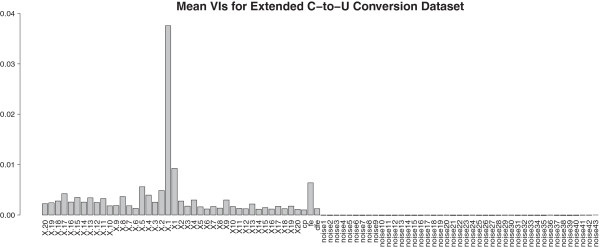
**Mean VIs for the 43 original predictors and 43 noise predictors from the balanced modified C-to-U conversion dataset.** Mean VIs were obtained by averaging the VIs (by commonly used error-rate-based permutation VIM) over 100 extended versions of the C-to-U conversion dataset.

Application of the standard permutation VIM to the data using the 2613 observations without missing values gave VIs greater than zero for all 43 predictors for different random seeds (i.e. different starting values for the random permutation), indicating that all predictors seem to have at least a small predictive power (data not shown). We generated and added additional predictors without any effect (termed noise predictors in the following) in order to evaluate the performance of error-rate-based and AUC-based permutation VIMs. Provided that there is a higher association between the response and any of the original predictors than between the response and any of the simulated noise predictors, a well performing VIM would attribute a higher VI to original predictors than to simulated noise predictors. The noise predictors were generated by randomly permuting the values of the original predictors. Each original predictor was permuted once, resulting in a total of 43 noise predictors. The whole process consisting of (1) creating 43 noise predictors, (2) merging them to the original dataset, (3) randomly subsampling to create an unbalanced dataset and (4) computing the error-rate-based and AUC-based permutation VIs, was repeated 100 times for each imbalance level to get stable results for the VIM performance. To check the assumption that there is a higher association between the response and any of the original predictors than between the response and any of the simulated predictors, we computed the mean VI over 100 completely balanced datasets that had been extended by noise predictors. Figure 1 shows that all mean VIs of the original predictors are higher than any mean VI of a simulated noise predictor and hence confirms our first impression.

### Performance evaluation criteria

VIMs give a ranking of the predictors according to their association with the response. To evaluate the quality of the rankings by the permutation VIMs the AUC was used as performance measure. The AUC was computed to assess the ability of a VIM to differentiate between associated predictors and predictors not associated with the response. AUC values of 1 mean that each associated predictor receives a higher VI than any noise predictor, thus indicating a perfect discrimination. AUC values of 0.5 mean that a randomly drawn associated predictor receives a higher VI than a randomly drawn noise predictor in only half of the cases, indicating no discriminative ability.

For our comparison studies we defined the two classes which are to be differentiated by a VIM in the following way. In the first instance of our studies on simulated data, all predictors which are associated with the response formed one class and noise predictors built the other class. In more detailed subsequent analyses we then explored the ability of the VIMs to discriminate between predictors with the same effect size and predictors without an effect. For this analysis one class comprised the noise predictors while the other class comprised only predictors with the same effect. For the studies on real data it was not possible to conduct such detailed analyses because the true ordering of the predictors according to their association with the response is not known. Hence in the analysis on real data we restricted our analysis to the discrimination between original predictors forming one class and simulated noise predictors forming the other class.

## Results and discussion

### Why may the error-rate-based permutation VIM fail in case of class imbalance?

The prioritisation of the majority class in unbalanced data settings is well known in the context of RF classification and can easily be seen from trees constructed on unbalanced data. Trees trained on unbalanced data more often predict the majority class, which leads to the minimization of the overall error rate. But how does this affect the performance of the permutation VIMs? And why is the AUC-based permutation VIM expected to be more robust towards class imbalance than the commonly used error-rate-based permutation VIM?

To answer these questions we consider an extremely unbalanced data setting and illustrate what happens in a tree when permuting the values of an associated predictor. We will first have a look at observations from the majority class. For this class nearly all observations are correctly classified by a tree which has been trained on extremely unbalanced data. If we now permute the values of an associated predictor, this does generally not result in a classification into the minority class since a classification into the minority class is an unlikely event – even for an observation from this class. A very specific data pattern is required for an observation to be classified into the minority class. It is unlikely that a random permutation of an associated predictor results in such a specific data pattern just by chance. Thus, for the majority class we expect hardly any observation to be incorrectly classified to the minority class after the permutation of an associated predictor. Thus the error rate does not considerably increase after the permutation of an associated predictor, finally leading to a rather low contribution to the VI.

Now let us consider the classifications by a tree for observations from the minority class. For an extreme class imbalance most of the observations from the minority class are falsely classified to the majority class due to the above described focus on the majority class. It might be the case that some observations from the minority class are correctly classified by the tree because these observations have that specific pattern of predictor values which is required for an observation to be classified into the minority class. It is likely that a permutation of the values of an associated predictor might then destroy that specific pattern so that after the permutation, these observations are not identified anymore to be in the minority class. Thus a misclassification due to the elimination of an associated predictor is much more likely to appear in observations from the minority class than in observations from the majority class. Note that only a small number of observations from the minority class are affected since most of the observations from the minority class are classified into the majority class anyway (before as well as after the permutation). The change in error rates is thus expected to be rather small – albeit it is more pronounced than the change in error rates in the majority class.

Note that the error-rate-based permutation VIM does not take class affiliations into account. Thus the change in error rates is actually not computed separately for each class. Yet, in order to better understand the behavior of the VIM, it may help to point out that if the class proportions were the same in all OOB samples, the VI of a predictor could be directly derived as the weighted average of the class specific differences in the error rates. The weights would correspond to the proportion of observations from the respective class. In practice the class frequencies will not be equal in all OOB samples, but the concept of a weighted average of the class specific error rates illustrates the fact that for unbalanced data settings the VI is mainly driven by the change in error rates derived from observations from the majority class. Since the change in error rates in the majority class is expected to be much smaller compared to the change in error rates in the minority class, the computed VIs are rather low. This results in low VIs even for associated predictors and in a poor differentiation of associated predictors and predictors not associated with the response.

### Class specific VIs

**Figure 2:**
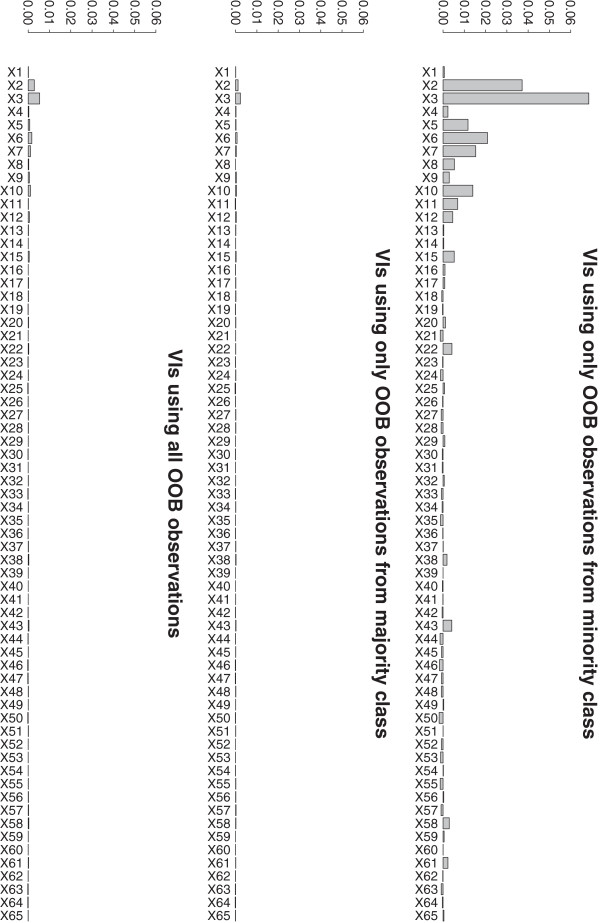
**VIs computed only from OOB observations of the minority class (top), from OOB observations of the majority class (middle) and from all OOB observations (bottom).** The first 15 predictors are associated with the response while the remaining predictors are noise predictors. VIs are shown for a total sample size of n = 500 and an imbalance level of 5%.

This theory is supported by computing class specific VIs (corresponding to mean changes in error rates computed only from observations belonging to the same class). Computing class specific VIs was done using the R package randomForest implementing the standard RF algorithm. The importance function of this package provides permutation VIs computed separately for each class (besides the VIs by the standard permutation VIM and by the Gini VIM). The class specific VIs for a total sample size of n = 500 and an imbalance level of 5% are shown in Figure 2, where predictors X_1_ to X_15_ have an effect while the remaining 50 predictors do not have an effect, corresponding to the simulation setting previously described in Table 1 in the context of the comparison study (for simplicity, we use the same setting as in the comparison study, although the addressed problem is here a different one). Different sample sizes and imbalance levels give similar results (thus not shown). They confirm our argumentation that the change in the error rates computed from OOB observations from the majority class is smaller than the change in error rates computed from OOB observations from the minority class. This results in an underestimation of the actual permutation VI due to a much higher weighting of the majority class in the computation of the VI (see concordance of VIs in middle and lower panel of Figure 2). The discrepancy between the VIs computed from observations of the minority class and VIs computed from observations of the majority class depends on the class imbalance and is more pronounced for more extreme class imbalances.

This motivates the use of an alternative accuracy measure which better incorporates the minority class. While the error rate gives the same weight to all observations, therefore focusing more on the majority class, the AUC is a measure which does not prefer one class over the other but instead puts exactly the same weight on both classes. Therefore the AUC-based permutation VIM is expected to detect changes in tree predictions for observations from the minority class, which might not be grasped by the error-rate-based permutation VIM due to a much higher weighting of the majority class. The VIs for associated predictors obtained by the AUC-based permutation VIM are thus expected to be comparatively higher than the VIs obtained by the error-rate-based permutation VIM. This would result in a better differentiation of associated and noise predictors by the AUC-based permutation VIM. These conjectures are assessed in the comparison study presented in the next section. (An additional performance comparison between the AUC-based permutation VIM and the error-rate-based permutation VIM based only on observations from the minority class is documented in Additional file 1.)

### Comparison study with simulated data

**Figure 3:**
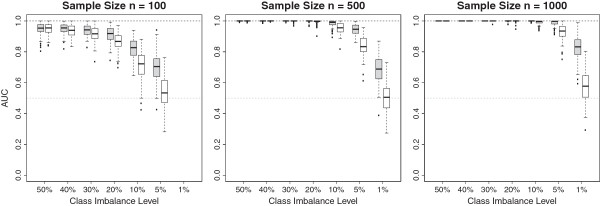
**Distribution of AUC-values for 100 simulated datasets for AUC-based (filled) and error-rate-based (unfilled) permutation VIMs for different class imbalances.** The AUC is used to assess the ability of a VIM to discriminate between predictors with an effect and predictors without an effect. Distributions are shown for total sample sizes of n = 100 (left panel), n = 500 (middle panel) and n = 1000 (right panel).

The performance of the error-rate-based and AUC-based VIMs as measured by the AUC is shown in Figure 3 for the three different total sample sizes with n = 100 (left panel), n = 500 (middle panel) and n = 1000 observations (right panel) and different class imbalance levels. Filled boxes correspond to the AUC-based permutation VIM and unfilled boxes correspond to the error-rate-based permutation VIM. Figure 3 shows that the performance of both VIMs decreases with an increasing class imbalance for all sample sizes. Note that the decrease in performance for both VIMs is not solely attributable to the imbalance ratio per se but also to the reduced number of observations in the minority class with an increasing class imbalance. This is induced by the simulation setting since we held the total number of observations fixed and varied the number of observations in both classes to create different class imbalances. If there are only few observations in one class then the tree predictions are less accurate. However the performance of the AUC-based permutation VIM decreases less dramatically than the performance of the error-rate-based permutation VIM. The discrepancy in performances between the VIMs increases with increasing imbalance level and is maximal for the most extreme class imbalance. While for a sample size of n = 500 the error-rate-based permutation VIM is no longer able to discriminate between associated and noise predictors (AUC values randomly vary around 0.5) for the most extreme class imbalance of 1%, the AUC-based permutation VIM still is, showing that it can be used to identify associated predictors even if the minority class comprises only few observations. It can be ruled out that the better performance of the AUC-based permutation VIM is due to chance since the distributions of AUC values significantly differ. Furthermore this difference in performances between both VIMs becomes even larger for larger sample sizes.

In a nutshell, in this first simulation the AUC-based permutation VIM performed better in case of class imbalance. The following subsections focus on the influence of sample size and effect size on the respective performance of both permutation VIMs in unbalanced data settings.

### Influence of sample size

In Figure 3, the performance of both VIMs improves with an increased total sample size for a fixed imbalance level since an increase in the sample size results in more accurate tree predictions. The right panel of Figure 3 shows that both permutation VIMs are hardly affected by class imbalances up to 10% when the sample size is rather large (n = 1000). If the sample size is smaller (n = 100), however, the performance of the VIMs is considerably decreased for a 10% imbalance level. A decrease in performance for a 10% imbalance level is also observed for a sample size of n = 500, especially for error-rate-based permutation VIM. In a nutshell, class imbalance seems to be more problematic for the permutation VIMs if the total sample size is small.

### Influence of effect size

**Figure 4:**
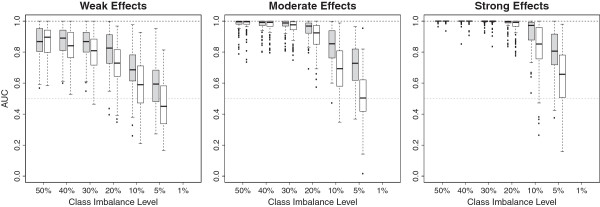
**Distribution of AUC-values for 100 simulated datasets for AUC-based (filled) and error-rate-based (unfilled) permutation VIMs for different class imbalances.** The AUC is used to assess the ability of a VIM to discriminate between noise predictors and predictors with a weak (left panel), moderate (middle panel) and strong (right panel) effect. Distributions are shown for a total sample size of n = 100.

We now explore the ability of the permutation VIMs to identify predictors with different effect sizes in presence of unbalanced data. The AUC was again used as an evaluation criterion to compare the ability of the AUC-based and error-rate-based permutation VIMs to discriminate between associated and non-associated predictors. Here the evaluation was done for each effect size separately meaning that one class comprised all the noise predictors while the other class comprised only predictors with the considered effect size (either strong, moderate or weak). Figure 4 shows the results for the setting with n = 100. The results for other sample sizes are shown in Additional file 2. The left panel of Figure 4 shows the performance of both permutation VIMs according to their ability to discriminate between predictors with weak effects and predictors without an effect. The middle panel corresponds to the AUC values for predictors with a moderate effect versus noise predictors and the right panel corresponds to the AUC values for predictors with a strong effect versus noise predictors.

Unsurprisingly, for both permutation VIMs predictors having only a weak effect are less discriminable from noise predictors than predictors with stronger effects. For imbalances up to 20% both VIMs identify nearly all predictors with a strong effect. Obviously there are unbalanced data settings where the standard permutation VIM still perfectly separates between noise predictors and predictors with pronounced effects. We conclude that class imbalance is more problematic if predictors with weak effects are to be identified while it plays a minor role if the classes are well separable.

### Comparison study with real data

**Figure 5:**
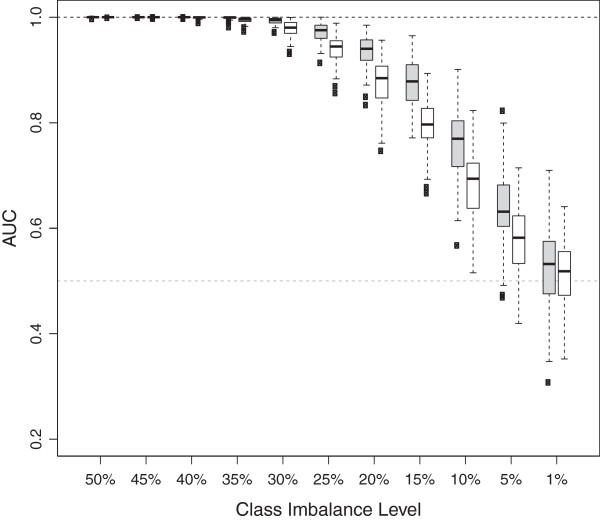
**Distribution of AUC-values for AUC-based (filled) and error-rate-based (unfilled) permutation VIMs for different class imbalances derived from 100 modified datasets from C-to-U conversion data.** The AUC is used to assess the ability of a VIM to discriminate between associated predictors and predictors not associated with the response.

Figure 5 shows the distribution of AUC values for 100 modified C-to-U conversion datasets for varying imbalance levels. For the balanced dataset and for slight class imbalances up to 40% both VIMs have a perfect discriminative ability since all associated predictors receive a higher VI than any noise predictor. Overall the performance of both VIMs decreases with an increasing class imbalance. Note that the decreasing performance for increasing class imbalances might be partly attributable to the reduced total sample size as the class imbalance was created by randomly subsampling observations from the class with the edited sites. When comparing both VIMs the AUC-based permutation VIM significantly outperformed the standard permutation VIM. For an imbalance of 30% the AUC-based permutation VIM clearly identified more associated predictors than the error-rate-based permutation VIM. The superiority of the AUC-based permutation VIM over the standard permutation VIM increased with an increasing class imbalance. For imbalances between 15% and 5% the discrepancy between the performance of AUC-based and standard permutation VIM was maximal.

Overall, this study on real data impressively shows that the AUC-based permutation VIM also works for complex real data and outperforms the standard permutation VIM in almost all class imbalance settings.

## Conclusions

The problem of unbalanced data has been widely discussed in the literature for diverse classifiers including random forests. Many approaches have been developed to improve the predictive ability of RF classifiers for unbalanced data settings. However less attention has been paid to the behaviour of random forests’ variable importance measures for unbalanced data. In this paper we explored the performance of the permutation VIM for different class imbalances and proposed an alternative permutation VIM which is based on the AUC.

Our studies on simulated as well as on real data show that the commonly used error-rate-based permutation VIM loses its ability to discriminate between associated predictors and predictors not associated with the response for increasing class imbalances. This is particularly crucial for small sample sizes and if predictors with weak effects are to be detected. The decreasing performance of the standard permutation VIM results from two sources: the class imbalance on the training data level leading to trees more often predicting the majority class and the class imbalance at the OOB data level leading to blurred VIs due to a much higher weighting of error rate differences in the majority class. A higher weighting of the majority class in the VI calculation is problematic because the difference in error rates is shown to be less pronounced in the majority class than in the minority class. Note that in some cases it might be interesting to assess the increase in error rate obtained when a certain predictor is removed. In this case the error-rate-based permutation VIM can be considered. If the goal is to rank the predictors according to their discrimination power, however, the AUC-based permutation VIM should be preferred.

The problem of imbalance at the OOB data level is directly addressed with the use of a novel AUC-based permutation VIM. This VIM puts the same weight on both classes by measuring the difference in AUCs instead of the difference in error rates. It is thus able to detect changes in tree predictions when permuting associated predictors which might not be grasped by the standard permutation VIM. In contrast, the imbalance on training data level is not addressed by the AUC-based permutation VIM, meaning that the structure of a tree remains untouched. On the one hand this is a drawback since class predictions before and after permuting a predictor are similar even if the respective predictor is associated with the response, resulting in a reduced change in the AUCs. On the other hand preserving the tree structure can be regarded as an advantage since a change in tree structure might open space for new unexpected behaviours. It is a major advantage of our novel AUC-based permutation VIM that it is based on exactly the same principle and differs from the standard permutation VIM only with respect to the accuracy measurement. It is thus expected to share the advantages of the standard permutation VIM and its properties and behaviours discovered in recent years (e.g. its behaviour in presence of correlated predictors [31] and in presence of predictors with different scales [22] and category sizes in the predictors [24,25]).

Our studies on simulated as well as on real data show that the AUC-based permutation VIM outperforms the commonly used error-rate-based permutation VIM as well as the error-rate-based permutation VIM computed only using observations from the minority class in case of unbalanced data settings (see Additional file 1 for the comparison to the class specific VIM). The difference in performance between our novel AUC-based permutation VIM and the standard permutation VIM can be substantial, especially for extremely unbalanced data settings. But even for slight class imbalances the AUC-based permutation VIM has shown to be superior to the standard permutation VIM. We conclude from our studies that the AUC-based permutation VIM should be preferred to the standard permutation VIM whenever two response classes have different class sizes and the aim is to identify relevant predictors.

### Availability and requirements

The AUC-based permutation VIM is implemented in the new version of the party package for the freely-available statistical software R (http://www.r-project.org and http://cran.r-project.org/web/packages/party/index.html). It can be applied via the function varimpAUC.

All codes implementing our studies on simulated and on real data are available under http://www.ibe.med.uni-muenchen.de/organisation/mitarbeiter/070_drittmittel/janitza/index.html for reproducibility purposes.

## Abbreviations

AUC: Area under curve; OOB: Out-of-bag; RF: Random forest; VIM: Variable importance measure; VI: Variable importance.

## Competing interests

The authors declare that they have no competing interests.

## Authors’ contributions

SJ wrote the paper and conducted all analyses. SJ and ALB developed and implemented the new VIM. All authors contributed to the design of the analyses and substantially edited the manuscript.

## Supplementary Material

Additional file 1 This file shows the results of the performance comparison between the AUC-based permutation VIM and the error-rate-based permutation VIM computed using only observations from the minority class. Click here for file

Additional file 2: **This file shows the distribution of AUC-values (analog to Figure** 4**) for sample sizes n = 500 and n = 1000.** (PDF 55 kb) Click here for file

## References

[B1] Breiman L Random forests Machine Learning 2001 45 5 32 10.1023/A:1010933404324

[B2] Boulesteix AL Janitza S Kruppa J König I Overview of random forest methodology and practical guidance with emphasis on computational biology and bioinformatics Wiley Interdisciplinary Reviews: Data Mining and Knowledge Discovery 2012 2 6 493 507 10.1002/widm.1072

[B3] Briggs F Goldstein B McCauley J Zuvich R De Jager P Rioux J Ivinson A Compston A Hafler D Hauser S Variation within DNA repair pathway genes and risk of multiple sclerosis Am J Epidemiol 2010 172 2 217 10.1093/aje/kwq086 20522537 PMC3658128

[B4] Chang J Yeh R Wiencke J Wiemels J Smirnov I Pico A Tihan T Patoka J Miike R Sison J Pathway analysis of single-nucleotide polymorphisms potentially associated with glioblastoma multiforme susceptibility using random forests Cancer Epidemiol Biomarkers Prev 2008 17 6 1368 1373 10.1158/1055-9965.EPI-07-2830 18559551 PMC6986563

[B5] Liu C Ackerman H Carulli J A genome-wide screen of gene–gene interactions for rheumatoid arthritis susceptibility Hum Genet 2011 129 5 473 485 10.1007/s00439-010-0943-z 21210282

[B6] Nicodemus K Callicott J Higier R Luna A Nixon D Lipska B Vakkalanka R Giegling I Rujescu D Clair D Evidence of statistical epistasis between DISC1, CIT and NDEL1 impacting risk for schizophrenia: biological validation with functional neuroimaging Hum Genet 2010 127 4 441 452 10.1007/s00439-009-0782-y 20084519

[B7] Sun Y Cai Z Desai K Lawrance R Leff R Jawaid A Kardia S Yang H Classification of rheumatoid arthritis status with candidate gene and genome-wide single-nucleotide polymorphisms using random forests BMC Proceedings 2007 1 Suppl 1 S62 10.1186/1753-6561-1-s1-s62 18466563 PMC2367463

[B8] Blagus R Lusa L Class prediction for high-dimensional class-imbalanced data BMC Bioinformatics 2010 11 523 10.1186/1471-2105-11-523 20961420 PMC3098087

[B9] Lin WJ Chen J Class-imbalanced classifiers for high-dimensional data Brief Bioinform 2012 10.1093/bib/bbs006 22408190

[B10] Khoshgoftaar T Golawala M Van Hulse J An empirical study of learning from imbalanced data using random forest Tools with Artificial Intelligence, 2007 2007 ICTAI 2007: 19th IEEE International Conference on, Volume 2, IEEE 310 317

[B11] Huang Y Hung C Jiau H Evaluation of neural networks and data mining methods on a credit assessment task for class imbalance problem Nonlinear Analysis: Real World Applications 2006 7 4 720 747 10.1016/j.nonrwa.2005.04.006

[B12] Fawcett T Provost F Adaptive fraud detection Data Mining and Knowledge Discovery 1997 1 3 291 316 10.1023/A:1009700419189

[B13] Kubat M Holte R Matwin S Machine learning for the detection of oil spills in satellite radar images Machine Learning 1998 30 2 195 215 10.1023/A:1007452223027

[B14] Chen C Liaw A Breiman L Using random forest to learn imbalanced data 2004 University of California, Berkeley: Tech. rep [http://statistics.berkeley.edu/tech-reports/666]

[B15] Xie Y Li X Ngai E Ying W Customer churn prediction using improved balanced random forests Expert Systems with Applications 2009 36 3 5445 5449 10.1016/j.eswa.2008.06.121

[B16] Batista G Prati R Monard M A study of the behavior of several methods for balancing machine learning training data ACM SIGKDD Explorations Newsletter 2004 6 20 29 10.1145/1007730.1007735

[B17] Estabrooks A Jo T Japkowicz N A multiple resampling method for learning from imbalanced data sets Computational Intelligence 2004 20 18 36 10.1111/j.0824-7935.2004.t01-1-00228.x

[B18] Van Hulse J Khoshgoftaar T Napolitano A Experimental perspectives on learning from imbalanced data 2007 ACM: In Proceedings of the 24th International Conference on Machine Learning 935 942

[B19] Van Hulse J Khoshgoftaar T Knowledge discovery from imbalanced and noisy data Data & Knowledge Engineering 2009 68 12 1513 1542 10.1016/j.datak.2009.08.00523573530

[B20] Japkowicz N Stephen S The class imbalance problem: A systematic study Intelligent Data Analysis 2002 6 5 429 449

[B21] Khalilia M Chakraborty S Popescu M Predicting disease risks from highly imbalanced data using random forest BMC Med Inform Decis Mak 2011 11 51 10.1186/1472-6947-11-51 21801360 PMC3163175

[B22] Strobl C Boulesteix AL Zeileis A Hothorn T Bias in random forest variable importance measures: Illustrations, sources and a solution BMC Bioinformatics 2007 8 25 10.1186/1471-2105-8-25 17254353 PMC1796903

[B23] Nicodemus KK Malley JD Predictor correlation impacts machine learning algorithms: implications for genomic studies Bioinformatics 2009 25 15 1884 1890 10.1093/bioinformatics/btp331 19460890

[B24] Nicodemus KK Letter to the editor: On the stability and ranking of predictors from random forest variable importance measures Brief Bioinform 2011 12 4 369 373 10.1093/bib/bbr016 21498552 PMC3137934

[B25] Boulesteix AL Bender A Bermejo JL Strobl C Random forest Gini importance favours SNPs with large minor allele frequency: assessment, sources and recommendations Brief Bioinform 2012 13 292 304 10.1093/bib/bbr053 21908865

[B26] Calle M Urrea V Boulesteix AL Malats N AUC-RF: A new strategy for genomic profiling with random forest Hum Hered 2011 72 2 121 132 10.1159/000330778 21996641

[B27] Hothorn T Hornik K Zeileis A Unbiased recursive partitioning: A conditional inference framework J Comput Graph Stat 2006 15 3 651 674 10.1198/106186006X133933

[B28] Pepe M The statistical evaluation of medical tests for classification and prediction 2004 USA: Oxford University Press

[B29] Hothorn T Hornik K Zeileis A Party: a laboratory for recursive partytioning 2012 R package version 0 3 URL http://cran.r-project.org/package=party

[B30] Cummings M Myers D Simple statistical models predict C-to-U edited sites in plant mitochondrial RNA BMC Bioinformatics 2004 5 132 10.1186/1471-2105-5-132 15373947 PMC521485

[B31] Nicodemus KK Malley J Strobl C Ziegler A The behavior of random forest permutation-based variable importance measures under predictor correlation BMC Bioinformatics 2010 11 110 10.1186/1471-2105-11-110 20187966 PMC2848005

