# Supplementary material for: An AUC-based permutation variable importance measure for random forests
Source: BMC Bioinformatics. 2013 Apr 5;14:119. doi: 10.1186/1471-2105-14-119 (PMC3626572; doi:10.1186/1471-2105-14-119)
Supplement: Additional file 1 — This file shows the results of the performance comparison between the AUC-based permutation VIM and the error-rate-based permutation VIM computed using only observations from the minority class. [file 1471-2105-14-119-S1.pdf]

Additional File 1

Performance comparison between the AUC-based permutation VIM and the error-rate-based permutation VIM computed using only observations from the minority class

I.

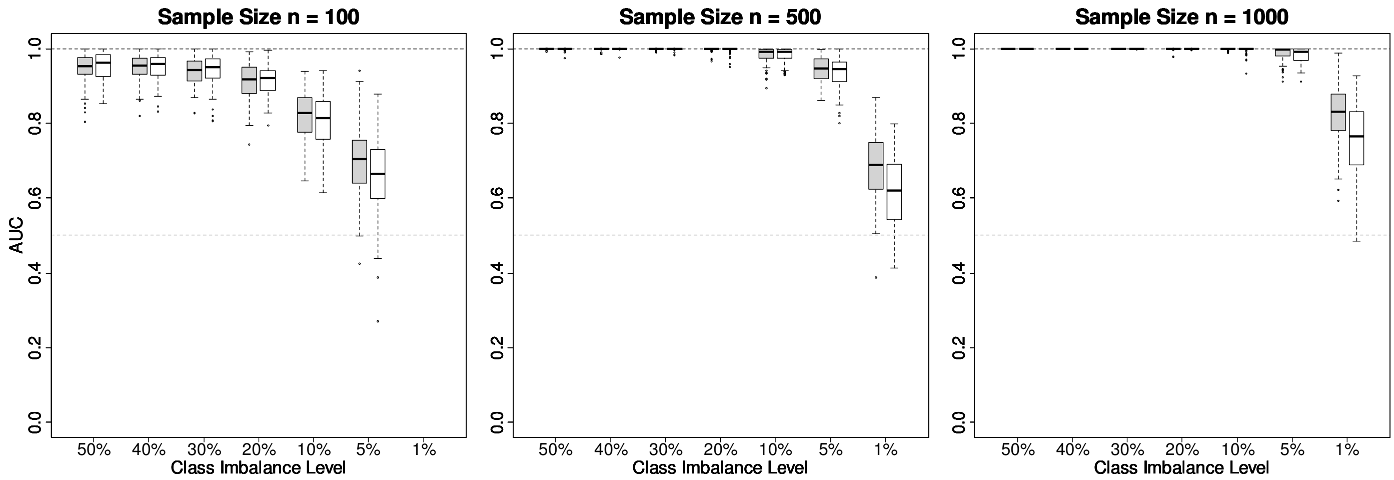

Distribution of AUC-values for 100 simulated datasets for AUC-based permutation VIM (filled) and error-rate-based permutation VIM computed using only observations from the minority class (unfilled) for different class imbalances. The AUC is used to assess the ability of a VIM to discriminate between predictors with an effect and predictors without an effect. Distributions are shown for total sample sizes of n = 100 (left panel), n = 500 (middle panel) and n = 1000 (right panel).

II.

(a) Sample Size n = 100

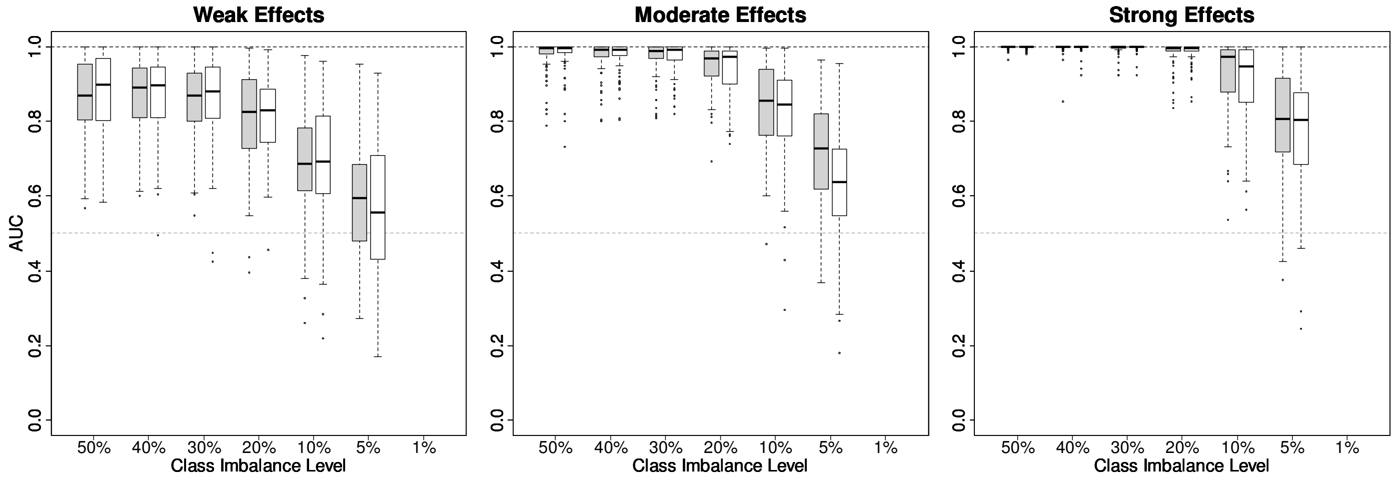

(b) **Sample Size n = 500**

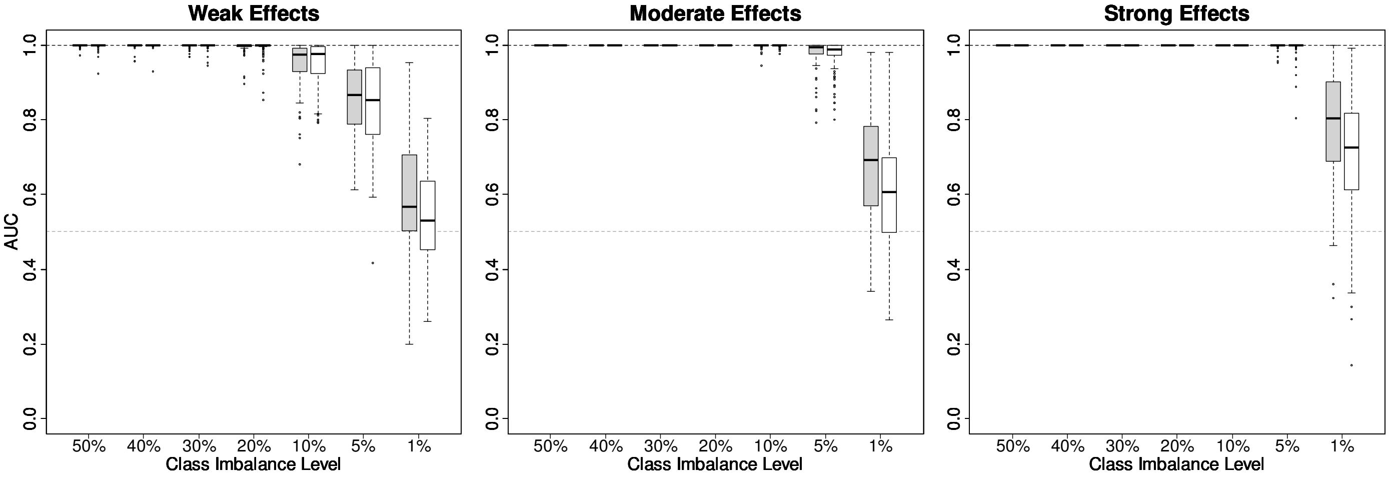

(c) **Sample Size n = 1000**

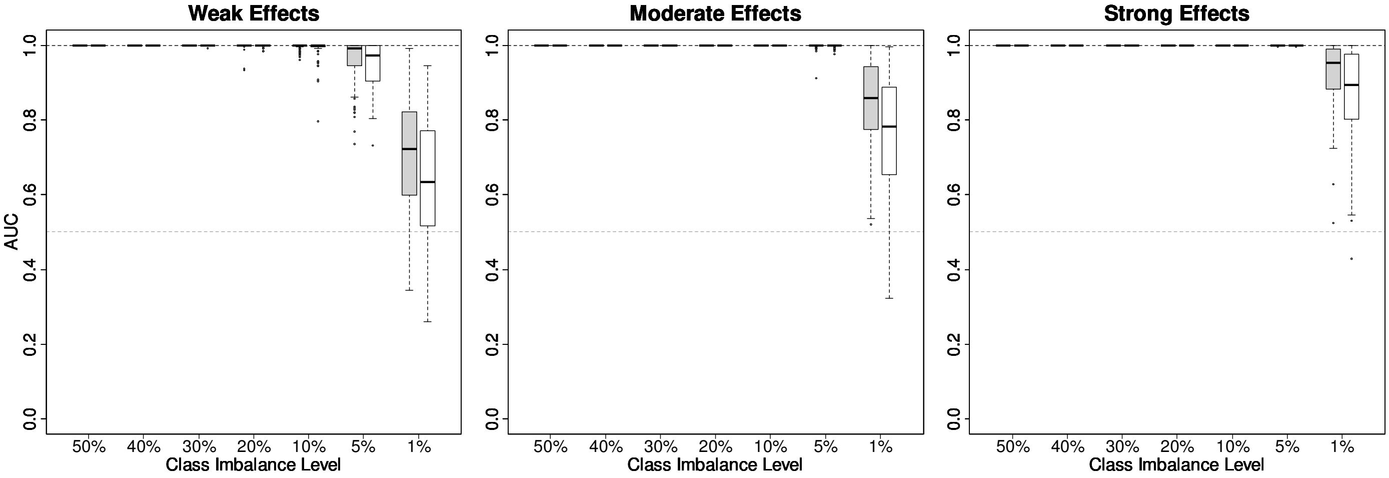

Distribution of AUC-values for 100 simulated datasets for AUC-based permutation VIM (filled) and error-rate-based permutation VIM computed using only observations from the minority class (unfilled) for different class imbalances. The AUC is used to assess the ability of a VIM to discriminate between noise predictors and predictors with a weak (left panel), moderate (middle panel) and strong (right panel) effect. Distributions are shown for a total sample size of **(a)** n = 100, **(b)** n = 500 and **(c)** n = 1000.
