# Supplementary material for: An AUC-based permutation variable importance measure for random forests
Source: BMC Bioinformatics. 2013 Apr 5;14:119. doi: 10.1186/1471-2105-14-119 (PMC3626572; doi:10.1186/1471-2105-14-119)
Supplement: Additional file 2: — This file shows the distribution of AUC-values (analog to Figure 4) for sample sizes n = 500 and n = 1000. (PDF 55 kb) [file 1471-2105-14-119-S2.pdf]

Additional File 2

Performance comparison between the AUC-based permutation VIM and the error-rate-based permutation VIM for sample sizes n = 500 and n = 1000

(a) **Sample Size n = 500**

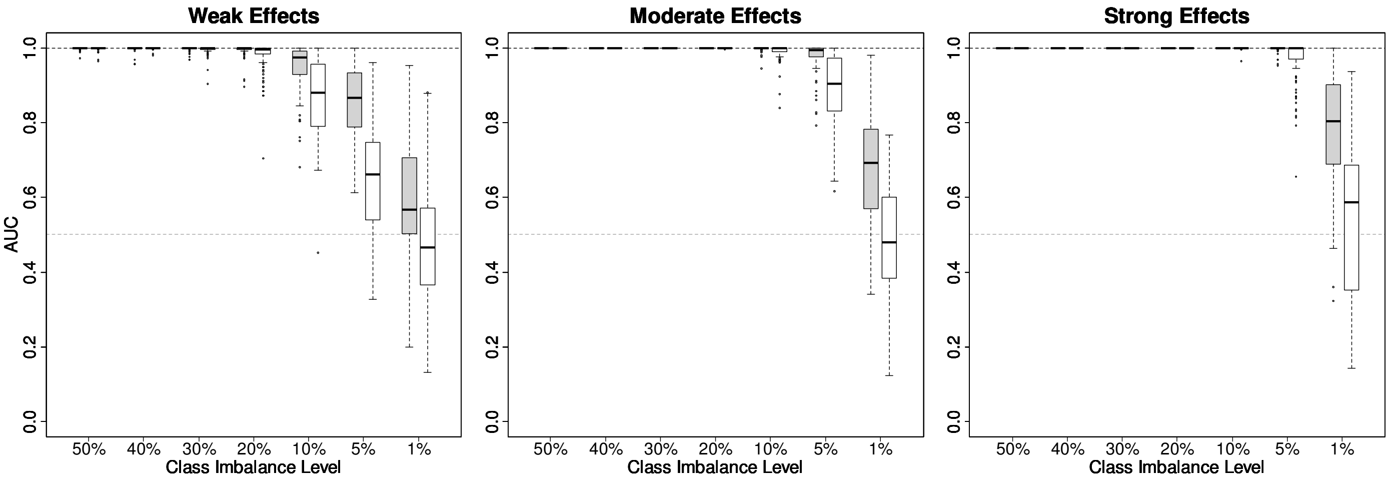

(b) **Sample Size n = 1000**

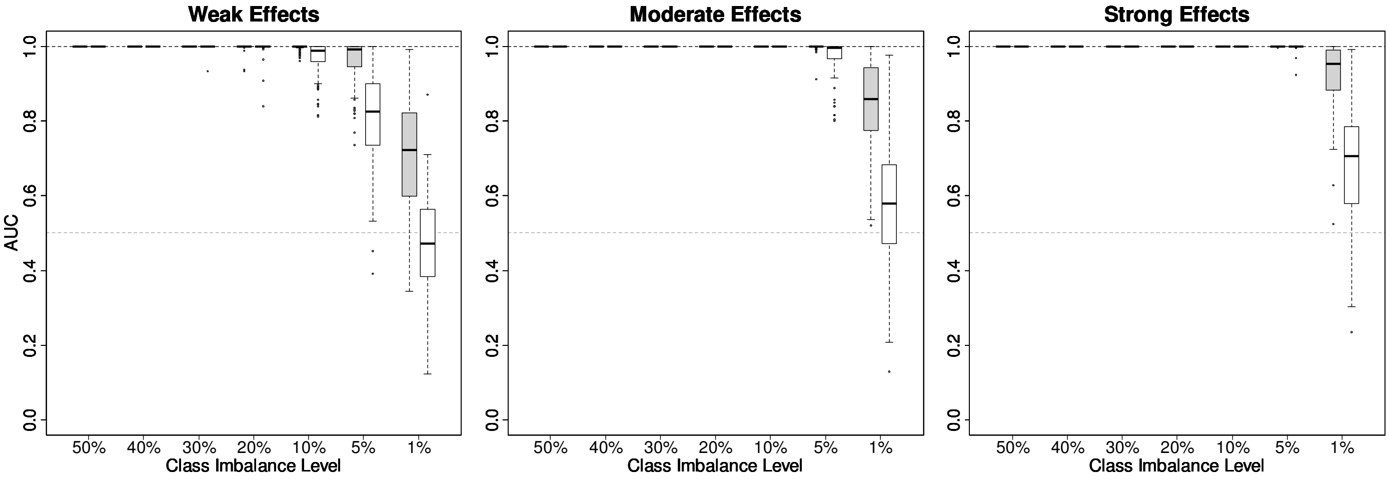

Distribution of AUC-values for 100 simulated datasets for AUC-based permutation VIM (filled) and error-rate-based permutation VIM (unfilled) for different class imbalances. The AUC is used to assess the ability of a VIM to discriminate between predictors with an effect and predictors without an effect. Distributions are shown for a total sample size of **(a)** n = 500 and **(b)** n = 1000.
